# Psychological status of general population 1 year after the outbreak of COVID-19: a cross-sectional study based on SCL-90

**DOI:** 10.3389/fpsyg.2024.1420834

**Published:** 2024-10-03

**Authors:** Xia Chen, Yue Hu, Yuan Deng, Xin Wang, Xiao Yang, Ying Wang, Yanli Lian, Shiping Wang, Xinju Xiang, Chan Liu, Fang Wu, Shaochuan Chen, Huimin Li

**Affiliations:** ^1^Neonatology Department, Chengdu Women’s and Children’s Central Hospital, School of Medicine, University of Electronic Science and Technology of China, Chengdu, China; ^2^Pediatrics Department, Chengdu Women’s and Children’s Central Hospital, School of Medicine, University of Electronic Science and Technology of China, Chengdu, China; ^3^Mammary Department, Chengdu Women’s and Children’s Central Hospital, School of Medicine, University of Electronic Science and Technology of China, Chengdu, China; ^4^Department of Nursing, Chengdu Women’s and Children’s Central Hospital, School of Medicine, University of Electronic Science and Technology of China, Chengdu, China; ^5^Obstetric Department, Chengdu Women’s and Children’s Central Hospital, School of Medicine, University of Electronic Science and Technology of China, Chengdu, China; ^6^Child Health Department, Chengdu Women’s and Children’s Central Hospital, School of Medicine, University of Electronic Science and Technology of China, Chengdu, China; ^7^Outpatient Department, Chengdu Women’s and Children’s Central Hospital, School of Medicine, University of Electronic Science and Technology of China, Chengdu, China

**Keywords:** COVID-19, epidemic, general population, mental health, SCL-90

## Abstract

**Introduction:**

The mental health of populations is usually affected after a disaster event. However, it is not known what the level of mental health of Chinese population 1 year after COVID-19, nor what factors influence it.

**Aim:**

This study aimed to examine the mental health status of general population in Chengdu 1 year after COVID-19, and then analyse influencing factors.

**Method:**

This study is a cross-sectional survey based on the SCL-90 questionnaire. Continuous data were described as M and SD, and counting data were described as frequencies(n) and percentages (%). Chi-square test or Fisher’s exact test were used for statistical inference, and significance variables were included in the binary logistic regression equation for multivariate analysis.

**Results:**

There were 172 participants with positive screening results. Age, marital status, number of kids, self-perceived health and the presence of chronic disease had an effect on screening results. Logistic regression analysis showed that age and self-perceived health were the main influencing factors.

**Discussion:**

Young people aged 18–19 and those who consider themselves not very healthy were at higher risk of poor mental health 1 year after the COVID-19 outbreak.

**Impact statement:**

Community institutions and community workers should focus on the mental health status of people 1 year after COVID-19, with a focus on people with poor self-perceived health and younger age groups, and take early preventive measures.

## 1 Introduction

Disaster events (such as earthquakes, hurricanes, outbreaks of epidemics, etc.) take a significant toll on human physical and mental health, and individuals affected by disasters are at risk of developing adverse mental health sequelae (Hu et al., 2021). COVID-19 has also been a disaster. On December 31, 2019, a new strain of coronavirus was isolated from patients with pneumonia of unknown etiology in Wuhan city, China, and named as severe acute respiratory syndrome coronavirus 2 (SARS-Cov-2) by the International Committee on Taxonomy of Viruses (ICTV) (Habas et al., 2020). On January 30, 2020, the World Health Organization declared that the COVID-19 outbreak is an international public health emergency, calling on all countries to take immediate action (World Health Organization, 2020). On March 11, 2020, WHO declared COVID-19 is a new pandemic (Anka et al., 2021).

The global excess mortality associated with COVID-19 was 14.91 million in the 24 months between 1 January 2020 and 31 December 2021, representing 9.49 million more deaths than those globally reported as directly attributable to COVID-19 (World Health Organization, 2022). The novel coronavirus has the characteristics of strong infectivity, multiple routes of infection, and wide spread. Since its full outbreak in December 2019, COVID-19 has become a global pandemic, causing a global public health crisis (He et al., 2020). COVID-19 is not only a threat to an individual’s physical health, it can also trigger mental health issues such as insecurity, fear and depression (Lindert et al., 2021).

With the progression of the outbreak, respiratory mucus droplets and direct contact have been identified as the main modes of human-to-human transmission. The basic strategies for the control of ongoing pandemic are dependent on the control policies and human behaviors, such as home isolation, contact tracing, social distancing, frequent handwashing (Habas et al., 2020). The implementation of these epidemic prevention measures may cause negative psychological reactions of general population, including adjustment disorders, anxiety disorders and depression, and then the psychological symptoms related to epidemics arise.

Researchers have found that after epidemics, such as SARS and MERS, the mental health of the population remained poor after one, two or even 3 years (Vindegaard and Benros, 2020). Moreover, a study has shown that the psychological symptoms of individuals were more severe 6 months after the epidemic than within 6 months (Yuan et al., 2021). This phenomenon is known as epidemic psychology. It is a unique field of research and applied science, suggests that the outbreak of an epidemic can have a significant impact on mental health (Taylor, 2022).

During COVID-19 pandemic a significant increase on COVID-19 Anxiety Syndrome (Alhakami et al., 2023; Mansueto et al., 2022), fatigue, loneliness (Mansueto et al., 2021) and a decrease in psychological flexibility and well-being has been observed across different countries (Mansueto et al., 2024; Carrozzino et al., 2021; Landi et al., 2020). A systematic review also showed that the general population experienced relatively high rates of symptoms of anxiety (6.33 to 50.9%), depression (14.6 to 48.3%), post-traumatic stress disorder (7 to 53.8%), psychological distress (34.43 to 38%) and stress (8.1 to 81.9%). Therefore, mitigating the adverse effects of COVID-19 on mental health has been recognized as a global public health priority (Xiong et al., 2020). But since the outbreak of COVID-19, researchers have paid much attention to understanding the epidemiology, clinical features, modes of transmission, resistance to virus transmission, and global health challenges, with limited attention to mental health of general population (Mukhtar, 2020).

Although previous studies have explored mental health status and related influencing factors of general population during the COVID-19 emergency response phase. They found that more than 70% of people had moderate or high level of psychological symptoms during this phase (Tian et al., 2020), but it remains unclear what the psychological status of general population 1 year after the outbreak of COVID-19. Based on a large number of studies on respiratory infectious diseases such as SARS and MERS, we hypothesized that general population will show a similar psychological trajectory after the outbreak of COVID-19 (Lung et al., 2009; Lee et al., 2007). That is to say, for a long period of time after the outbreak of COVID-19, the mental health of individuals may be poor, which will seriously affect the normal life of individuals. However, there is very little research to support our conjecture. Therefore, this study aims to investigate the psychological status of the general population 1 year after COVID-19 and to analyse the influencing factors. The results of this survey can provide professionals with a reference for early intervention, and provide support for the further development of epidemiological psychology.

## 2 Methods

### 2.1 Study design

This was an observational study and had been registered at the Clinical Trials Center. This study was in line with the Declaration of Helsinki revised in 2013 and received approval from the Medical Ethics Committee of Chengdu Women’s and Children’s Central Hospital (approval number: 2021(16)). Before participants start filling out the questionnaire, the researcher introduced the purpose and significance of the survey to them, and all participants were agreed to participate in the study.

### 2.2 Population and sample

Convenience sampling method was used to recruit subjects. The inclusion criteria were as follows: ① participants were ≥ 18 years old; ② ability to complete the survey using smartphones; ③ were informed about the purpose of the study; ④volunteered to participate. The exclusion criteria were as follows: ① illiterate; ② cannot understand the content of the questionnaire; ③ cannot complete the questionnaire independently. In the end, a total of 2,235 people were recruited in the survey.

### 2.3 Data collection

This was an online survey, and all participants used Wenjuanxing, a professional Chinese questionnaire survey platform, to complete the questionnaire. The first part of the questionnaire was informed consent form. Participants would first read the informed consent of the study, tick “agree” and then enter the questionnaire filling interface; otherwise, the survey would be finished.

### 2.4 Instrument

#### 2.4.1 Demographic and health related questionnaire

A self-designed questionnaire was used to collect demographic and health related information of the participants, including items for gender, age, marital status, number of kids, occupation, education background, any chronic illness, and perceived health status.

#### 2.4.2 Symptom Checklist 90 (SCL-90)

The 90-item symptom list (SCL-90), also known as the symptom self-rating scale, is the most widely used outpatient examination scale for mental disorders and mental illnesses. It was compiled by L.R. Derogatis in 1975 and suitable for adults over 16 years old. This scale can assess whether an individual has a certain psychological symptom and its severity from multiple perspectives (Tang et al., 1999). The SCL-90 scale has been translated into multiple languages and used in several countries around the world. It was introduced into mainland China in 1984 to study psychiatric symptoms. Then Chinese national norms was subsequently established for the first time. From then on, it has been widely used in general population surveys and large-scale psychological status screening research in China (Tan et al., 2015; Tsai et al., 2003; Dang et al., 2021). Data were collected using SCL-90 Chinese version, which demonstrated high reliability (Cronbach’s *α* of this scale was 0.98, Cronbach’s α of each factor score ranged from 0.80 to 0.91) (Yu et al., 2019), and the validity of the scale was 0.963, indicating that the scale had good reliability and validity, and could accurately reflect the mental health status of residents (Shi et al., 2013).

The SCL-90 includes nine subscales involving nine symptom dimensions, which are somatization (SOM), obsessive-compulsive disorder (OC), interpersonal sensitivity (IS), depression (DEP), anxiety disorder (ANX), hostility (HOS), phobic anxiety disorder (PHOB), paranoid perception (PAR), and psychosis (PSY), and the remaining seven items reflecting sleep and diet were listed as other dimensions. Nine subscales provided symptom descriptions, and participants assessed the symptoms described by the scale, with 1 for no symptoms, 2 for mild symptoms, 3 for moderate symptoms, 4 for severe symptoms and 5 for very serious symptoms. Participants was required to make an independent self-assessment based on their actual feelings of “now” or “the last week”. The main scoring indicators include the total score of 90 items;GSI (Global Severity Index) score: actual total score of the scale/90; factor score: total factor score/number of factor items; PST (Positive Symptom Total): the number of items with a single score ≥ 2; PSDI(Positive Symptom Distress Index): the total score of positive items/number of positive items (Gomez et al., 2021).

### 2.5 Data analysis

Data were analyzed by Statistical Package for the Social Sciences (IBM SPSS 26.0). Frequencies (n) and percentages (%) were used to describe general information include gender, age distribution, vaccination, marital status, number of kids, educational background, occupation, perceived health status, any chronic diseases. Shapiro-Wilktest was used to test the normality of the data. Continuous data were described as mean (M) and standard deviation (SD). To identify the differences in positive and negative SCL-90 screening groups according to demographic characteristics, Chi-square test and Fisher’s exact test were performed. Significant variables were incorporated into the binary logistic regression equation for multivariate analysis.

## 3 Results

### 3.1 General information

As shown in Table 1, a total of 2,235 people completed the survey, of which 906 (40.50%) were male, 1,329 (59.50%) were female;1,131 (50.60%) got one vaccination, and 1,104 (49.40%) got two vaccinations. Most of the respondents were over 30 years old (1,828, 81.79%), married (1,723, 77.10%), had one child (1,299, 58.10%), and had a college or university degree (1,001, 44.80%), engaged in business services (590, 26.40%), and considered themselves healthy (1,899, 85%), and with no chronic disease (1,944, 87.00%).

**Table 1:** General information of the sample population of this study.

Variables		*N*	%
Gender	Male	906	40.50%
	Female	1,329	59.50%
Age distribution (year)	18–19	29	1.30%
	20–29	378	16.90%
	30–39	622	27.80%
	40–49	552	24.70%
	≥50	654	29.30%
Vaccination	Once	1,131	50.60%
	Twice	1,104	49.40%
Marital status	Unmarried	355	15.90%
	Married	1,723	77.10%
	Divorced	136	6.10%
	Widowed	21	0.90%
Number of kids	0	427	19.10%
	1	1,299	58.10%
	2 or more	509	22.80%
Educational background	Junior middle school and below	611	27.30%
	High school	575	25.70%
	College degree or Bachelor	1,001	44.80%
	Master’s degree or above	48	2.10%
Occupation	Government organs and institutions	354	15.80%
	Professional skill worker	348	15.60%
	Business service industry	590	26.40%
	Agriculture, forestry, fishery and animal husbandry	85	3.80%
	Production and transportation	62	2.80%
	Soldier	9	0.40%
	Unemployed	368	16.50%
	Other	419	18.70%
Perceived health status	Healthy	1,899	85.00%
	Not very healthy	336	15.00%
Any chronic diseases	No	1,944	87.00%
	Yes	291	13.00%

The scores of each factor and total score of SCL-90 are shown in Table 2. DEP symptoms was the highest (14.54 ± 3.556), followed by SOM symptoms (13.22 ± 2.282), and PAR symptoms was the lowest (6.41 ± 1.223).

**Table 2:** SCL-90 scores for each factor in this study.

Factor	Minimum (point)	Maximum (point)	Mean score	SD
SOM	12	41	13.22	2.282
OC	10	35	11.94	3.357
IS	9	31	9.95	2.319
DEP	13	43	14.54	3.556
ANX	10	37	10.81	2.098
HOS	6	24	6.69	1.584
PHOB	7	23	7.37	1.209
PAR	6	21	6.41	1.223
PSY	10	32	10.73	2.057
Other	7	24	8.32	2.296
Total	90	268	100.00	18.792

SOM, somatization; OC, obsessive-compulsive disorder; IS, interpersonal sensitivity; DEP, depression; ANX, anxiety disorder; HOS, hostility; PHOB, phobic anxiety disorder; PAR, paranoid perception; PDY, psychosis.

**Figure 1:**
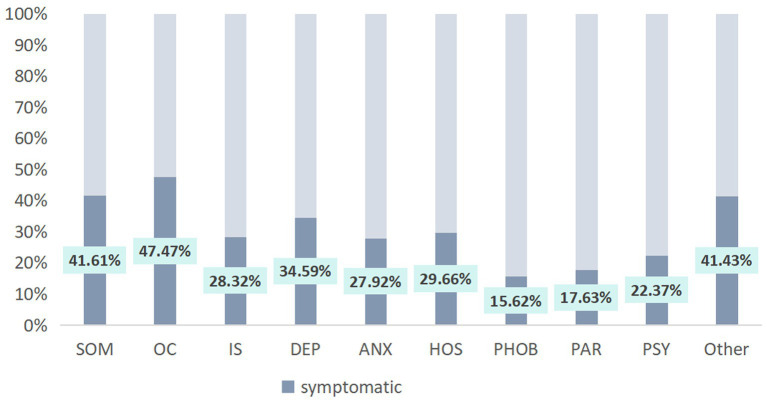
The proportion of symptomatic populations with SCL-90 factor scores in the sample population of this study. SOM, somatization; OC, obsessive-compulsive disorder; IS, interpersonal sensitivity; DEP, depression; ANX, anxiety disorder; HOS, hostility; PHOB, phobic anxiety disorder; PAR, paranoid perception; PDY, psychosis.

**Figure 2:**
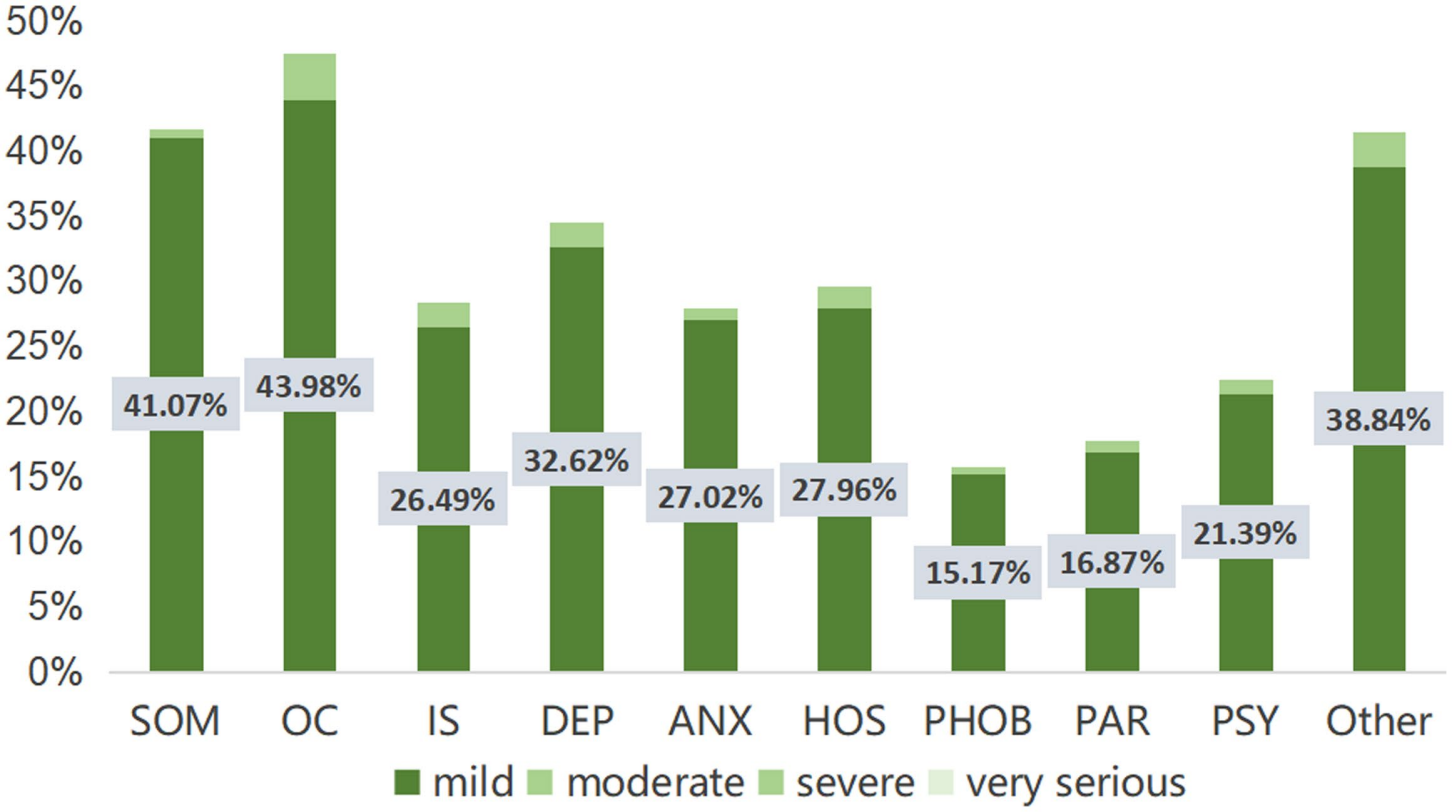
The severity distribution of SCL-90 factor scores in the sample population of this study. SOM, somatization; OC, obsessive-compulsive disorder; IS, interpersonal sensitivity; DEP, depression; ANX, anxiety disorder; HOS, hostility; PHOB, phobic anxiety disorder; PAR, paranoid perception; PDY, psychosis.

Table 3 shows the symptom severity of each factor score and total score of the study population. From the GSI score, 33.96% of the population were asymptomatic, 65.10% had mild symptoms, 0.94% had moderate symptoms, and no one had severe symptoms or very serious symptoms. In the distribution of symptom severity of each factor, the number of population with OC symptoms (1,061 people, 47.47%), SOM symptoms (930 people, 41.61%) and other symptoms (926 people, 41.43%) ranked the top three. Among the population with OC symptoms, 92.65% (983 people) had mild symptoms, 6.60% had moderate symptoms, and 0.75% had severe symptoms. Among population with SOM symptoms, 98.71% (918 people) had mild symptoms, 1.18% had moderate symptoms, and 0.11% had severe symptoms. Among the population with other symptoms, 93.74% (868 people) had mild symptoms, 5.40% had moderate symptoms, and 0.86% had severe symptoms. Figure 1 shows the distribution of SCL-90 scores of symptomatic and asymptomatic people in the sample of this study. Figure 2 shows the proportion of the population with SCL-90 score symptom severity in this study sample.

**Table 3:** Severity distribution of SCL-90 factor scores and GSI scores in the sample population of this study.

Factor	Asymptomatic		Symptomatic		1 < i ≤ 2 mild		2 < i ≤ 3 moderate		3 < i ≤ 4 severe		4 < i ≤ 5 very serious	
SOM	1,305	58.39%	930	41.61%	918	41.07%	11	0.49%	1	0.04%	0	0.00%
OC	1,174	52.53%	1,061	47.47%	983	43.98%	70	3.13%	8	0.36%	0	0.00%
IS	1,602	71.68%	633	28.32%	592	26.49%	39	1.74%	2	0.09%	0	0.00%
DEP	1,462	65.41%	773	34.59%	729	32.62%	40	1.79%	4	0.18%	0	0.00%
ANX	1,611	72.08%	624	27.92%	604	27.02%	17	0.76%	3	0.13%	0	0.00%
HOS	1,572	70.34%	663	29.66%	625	27.96%	33	1.48%	5	0.22%	0	0.00%
PHOB	1886	84.38%	349	15.62%	339	15.17%	8	0.36%	2	0.09%	0	0.00%
PAR	1841	82.37%	394	17.63%	377	16.87%	15	0.67%	2	0.09%	0	0.00%
PSY	1735	77.63%	500	22.37%	478	21.39%	20	0.89%	2	0.09%	0	0.00%
Other	1,309	58.57%	926	41.43%	868	38.84%	50	2.24%	8	0.36%	0	0.00%
GSI	759	33.96%	1,476	66.04%	1,455	65.10%	21	0.94%	0	0.00%	0	0.00%

i refers to the dimension score.

### 3.2 Univariate analysis of negative and positive groups of participants

If the total SCL-90 score exceeds 160 points, or the number of positive items exceeds 43, or any factor score exceeds 2 points, we consider the screening result to be positive for psychological status. In this study,172 participants (7.70%) were positive. As shown in Table 4, there was a significant difference in the psychological status of gender (*p* = 0.027), age (*p* < 0.001), marital status (*p* < 0.001), number of kids (*p* < 0.001), perceived health status (*p* < 0.001), and whether have chronic diseases (*p* = 0.012).

**Table 4:** The sample population of this study SCL-90 screening negative and positive groups chi-square test.

Variables		Negative		Positive		Test value	*P* value
		*N*	%	*N*	%		
Gender	Male	850	93.80%	56	6.20%	4.921	0.027
	Female	1,213	91.30%	116	8.70%		
Age (year)	18–19	22	75.90%	7	24.10%	32.493	<0.001
	20–29	329	87.00%	49	13.00%		
	30–39	572	92.00%	50	8.00%		
	40–49	528	95.70%	24	4.30%		
	50–59	612	93.60%	42	6.40%		
Vaccination	Once	1,039	91.90%	92	8.10%	0.62	0.431
	Twice	1,024	92.80%	80	7.20%		
Marital status	Unmarried	304	85.60%	51	14.40%	24.863	<0.001
	Married	1,616	93.80%	107	6.20%		
	Divorced	124	91.20%	12	8.80%		
	Widowed	19	90.50%	2	9.50%		
Number of kids	0 child	369	86.40%	58	13.60%	26.081	<0.001
	1 child	1,220	93.90%	79	6.10%		
	2 or more	474	93.10%	35	6.90%		
Educational background	Junior middle school and below	571	93.50%	40	6.50%	3.834	0.272
	High school	536	93.20%	39	6.80%		
	College degree or Bachelor	912	91.10%	89	8.90%		
	Master’s degree or above	44	91.70%	4	8.30%		
Occupation	Government organs and institutions	327	92.40%	27	7.60%	3.981	0.758
	Professional skill worker	321	92.20%	27	7.80%		
	Business service industry	547	92.70%	43	7.30%		
	Agriculture, forestry, fishery and animal husbandry	78	91.80%	7	8.20%		
	Production and transportation	59	95.20%	3	4.80%		
	Soldier	8	88.90%	1	11.10%		
	Unemployed	344	93.50%	24	6.50%		
	Other	379	90.50%	40	9.50%		
Perceived health status	Healthy	1,790	94.30%	109	5.70%	68.026	<0.001
	Not very healthy	273	81.20%	63	18.80%		
Have chronic diseases	No	1,805	92.80%	139	7.20%	6.256	0.012
	Yes	258	88.70%	33	11.30%		

### 3.3 Influencing factors analysis of negative and positive groups of participants

The significant variables of univariate analysis were included in the binary logistic regression model, and the forward stepwise regression method was used for analysis. *p* < 0.05 in The Omnibus Tests of Model Coefficients test, indicating that the model established in this study has statistical significance (χ^2^ = 90.392, *p* < 0.001). *p* = 0.901 > 0.05 in the Hosmer and Lemeshow Test, indicating that the model fits well. Nagelkerke *R^2^* can be used to evaluate the fit of the regression equation, *R^2^* takes a value between 0 to 1, the larger its value, the better the fit of the regression model. The value of *R^2^* is affected by the number of independent variables, and an increase in the number of independent variables increases the value of *R^2^*. The Nagelkerke *R^2^* of this model is 0.095, the lower *R^2^* may be related to the smaller number of independent variables in the model. In conclusion, this model has a good judgment effect. The risk of mental health problems of young people aged 18–19 years was 3.861 times that of individuals aged 30–39 years (*OR = 0.259, 95% CI:0.103 ~ 0.656*; *p = 0.004*); 7.407 times that of 40–49 year old individuals (*OR = 0.135, 95% CI:0.051 ~ 0.358; P<0.001*); and 5.814 times than that of residents aged 50 or older (*OR = 0.172, 95% CI:0.067 ~ 0.439*; *P<0.001*). Individuals who consider themselves not very healthy are 4.182 times more likely to have psychological symptoms than those who consider themselves healthy (*OR = 4.182, 95% CI:2.957 ~ 5.913*; *P<0.001*) (Table 5).

**Table 5:** Analysis of influencing factors of SCL-90 screening negative and positive groups in the sample population of this study.

Variables		*B*	SE	Wald	*p* value	OR	95% CI
Perceived health status	Healthy	Reference					
	Not very healthy	1.431	0.177	60.644	<0.001	4.182	2.957 ~ 5.913
Age	18–19	Reference					
	20–29	−0.761	0.475	2.570	0.109	0.467	0.184 ~ 1.185
	30–39	−1.349	0.473	8.132	0.004	0.259	0.103 ~ 0.656
	40–49	−1.999	0.496	16.254	<0.001	0.135	0.051 ~ 0.358
	≥50	−1.762	0.479	13.535	<0.001	0.172	0.067 ~ 0.439
(Constant)		−1.395	0.450	9.630	0.002	0.248	–

## 4 Discussion

The COVID-19 pandemic has brought unprecedented psychological stress to people around the world (Carlos et al., 2020; Levkovich and Shinan-Altman, 2021). This study examined the mental health status of general population in Chengdu, China. We found that 1 year after the outbreak, most people screened for SCL-90 have mild symptoms. Most importantly, we found that perceived health status and age were important influences on mental health. Poor subjective health status and younger age (18–19 years old) were independent risk factors for poorer mental health status.

**Self-perceived health (SPH)** is a subjective expression of health, which is widely used in population health research (Gumà, 2021). Perceived health status, also known as subjective health perception, represents the self-evaluation of an individual’s general health status. It refers to people’s overall perception of their own health status, including physical and psychological factors (Denche-Zamorano et al., 2022; Kwak et al., 2022). In this study, SPH was measured by ‘How do you usually view your health?’, and the responses were categorized as “healthy” or “not very healthy.” We found that the SPH status was the main influencing factor of community residents’ mental health, and community residents who self-perceived as unhealthy were more likely to have mental health problems. The results obtained are consistent with that reported by Inbar and Shinan-Altman (2021) and Buneviciene et al. (2022), suggesting that poorer perceived health is associated with increased risk of mental health problems. This phenomenon also may be explained by the concept of positive psychology. SPH is a positive emotion, it can improve the psychological state and make people’s psychology tend to be in a healthy state (Seligman et al., 2005). de-Mateo-Silleras et al. (2019) conducted a cross-sectional survey of 214 university students in Spain, the aim of the study was to assess their perception of health based on their lifestyle. The results of this study found that for the university population, a healthy lifestyle had a significant impact on health perception (de-Mateo-Silleras et al., 2019). The results of Cau et al. (2016) and Szwarcwald et al. (2015) also found that health behaviors, such as not smoking, consuming enough recommended amounts of fruits and vegetables, and engaging in physical exercise, can help to improve health perception, which in turn improves psychological status. So we believe that community residents can be encouraged to adopt a healthy lifestyle and do more exercise to improve their health perception and regulate their mental health level. Studies by D'Oliveira et al. (2022) and da Cruz et al. (2022) have concluded that physical activity improves the mental health of the population. D'Oliveira et al. (2022) conducted a study on a physical exercise protocol for older adults, applied remotely during the pandemic, this study offers a home-based exercise protocol for older adults. In line with the findings of D'Oliveira et al. (2022), our study also highlights the importance of adapted physical exercise protocols for vulnerable populations during periods of social isolation. Moreover, community agency organizations can use digital interventions to raise awareness of healthy lifestyles among general population. For example, mass media can be used to publicise the importance of a healthy lifestyle for the body and mind, what a healthy lifestyle is and how to choose a healthy lifestyle.

**Age** is an influential factor for mental health during COVID-19, which is consistent with the findings by Huang and Zhao (2020) and Chew et al. (2020), but differ in Inbar and Shinan-Altman (2021). Huang and Zhao (2020) collected data from 7,236 volunteers, found that the incidence of mental health problems in younger people was significantly higher than in older adults. But in another study conducted in Israel by Inbar and Shinan-Altman (2021) explored the relationship between emotional reactions and subjective health status during the COVID-19 pandemic, showing a high prevalence of emotional reactions among older adults. Despite inconsistent findings, most research now agrees that young people are more likely to have mental health problems.

The results of a recently published meta-analysis showed that mental health problems were most prominent among young people during COVID-19 (Dragioti et al., 2022). In another review, researchers also found that psychiatric symptom problems were prominent among young people (university students), which is consistent with our findings (Manchia et al., 2022). In our study, age is a protective factor, the older the age, the less likely the mental health problems are. On the contrary, the more likely the mental health problems are. Our study found that young people aged 18 to 19 years were more likely to have mental health problems 1 year after the outbreak of the epidemic. Possible explanations for this result are as follows: (a) young people have less life experience, less mental resilience, and are unable to recover in time after experiencing stressful events (Manchia et al., 2022); (b) residents aged 18–19 years are at the peak period for mental health problems. Exposure to the COVID-19 pandemic during this fragile developmental period may leave young people more vulnerable to the negative psychological effects of such events, and at high risk of negative psychological experiences followed by mental health problems (O'Reilly et al., 2021). Furthermore, researchers have shown that family support has an important impact on mental health (El Haj et al., 2020; Bethell et al., 2021). In China, most 18- and 19-year-olds are leaving their families and going to university alone, with limited family support in their daily lives, which can make them vulnerable to mental health problems. During the COVID-19 pandemic, due to the adoption of epidemic prevention measures such as home isolation and social reduction, the loneliness of young people was particularly prominent, while the increase of loneliness was an important reason for the emergence of mental health problems (Lee et al., 2020). So we believe that for young people aged 18–19, schools or communities should give them more support, such as holding group activities, reading salons, family days to enrich their daily lives. Furthermore, more attention needs to be paid to them, with the aim of early detection of mental health problems in young people and early intervention.

### 4.1 Implications for mental health nursing practice and research

In recent years, there has been an increasing focus on mental health. Mental health, as a sustainable development goal, plays an important role in achieving global development. The effects of poor mental health cover many areas of an individual’s life. Nursing approach to intervention required due to the significant impact on mental health in COVID-19 (Moitra et al., 2023). This study found that SPH and age were significant influences on mental health in general population following the COVID-19 epidemic. SPH is a modifiable factor. In the future, after a disaster event, the relevant personnel can intervene early in the mental health of the general population by adjusting their perceived state of illness, in order to prevent serious mental health problems. Age is an unmodifiable factor. Young people’s mental health problems are prominent after a disaster event, and require focused attention from professionals. Therefore, schools and communities and other relevant authorities need to consider early intervention and sustained attention to young people after a disaster event. The findings of these results also provide some ideas for future post-pandemic psychological care: (i) community nurses may play an important role in a pandemic, so their latent capacity needs to be recognized and stimulated; and (ii) coordinated care approaches need to be proposed in order to deal with post-pandemic mental health conditions; (iii) Young people are a vulnerable group with mental health problems and need more attention and early intervention.

In terms of research implications, future research could continue to focus on the mental health of the general population after COVID-19. Long-term follow-up studies may help us to understand the trajectory of mental health development in the general population after a disaster event. In addition, large-scale research studies could be conducted in the future to increase the representativeness of the sample population.

### 4.2 Limitations

As far as we know, this is the first study in China to investigate the mental health status of the general population 1 year after COVID-19. This study contributes to the development of epidemiological psychology. However, it has several limitations. First, this research is a cross-sectional survey, the absence of long-term follow-up limits the ability to determine the persistence of the observed effects. This is an important area for improvement in future research. Second, no information was collected about currently or previous pharmacological (e.g., characteristic of of BZD of SRRI) (Cosci et al., 2016) or psychological treatments (Swartz, 2020), although it may affect SCL-90 symptoms severity; Third, most of the study participants were community residents near a large tertiary hospital, with relatively few participants from other administration area, it might be more persuasive if it covered residents of more administrative districts in the city of Chengdu.

## 5 Conclusion

Based on the results of this study, 1 year after COVID-19, 7.70% of the general population in Chengdu still had psychosomatic symptoms. Poor self-perceived health and younger age were the main influencing factors affecting their mental health. After the outbreak, community agency organizations can take some measures for preventive intervention and monitoring of this population.

## Data Availability

The raw data supporting the conclusions of this article will be made available by the authors, without undue reservation.
